# Supplementary figures and images for: Current practices of management of maternal and congenital Cytomegalovirus infection during pregnancy after a maternal primary infection occurring in first trimester of pregnancy: Systematic review
Source: PLoS One. 2021 Dec 3;16(12):e0261011. doi: 10.1371/journal.pone.0261011 (PMC8641894; doi:10.1371/journal.pone.0261011)

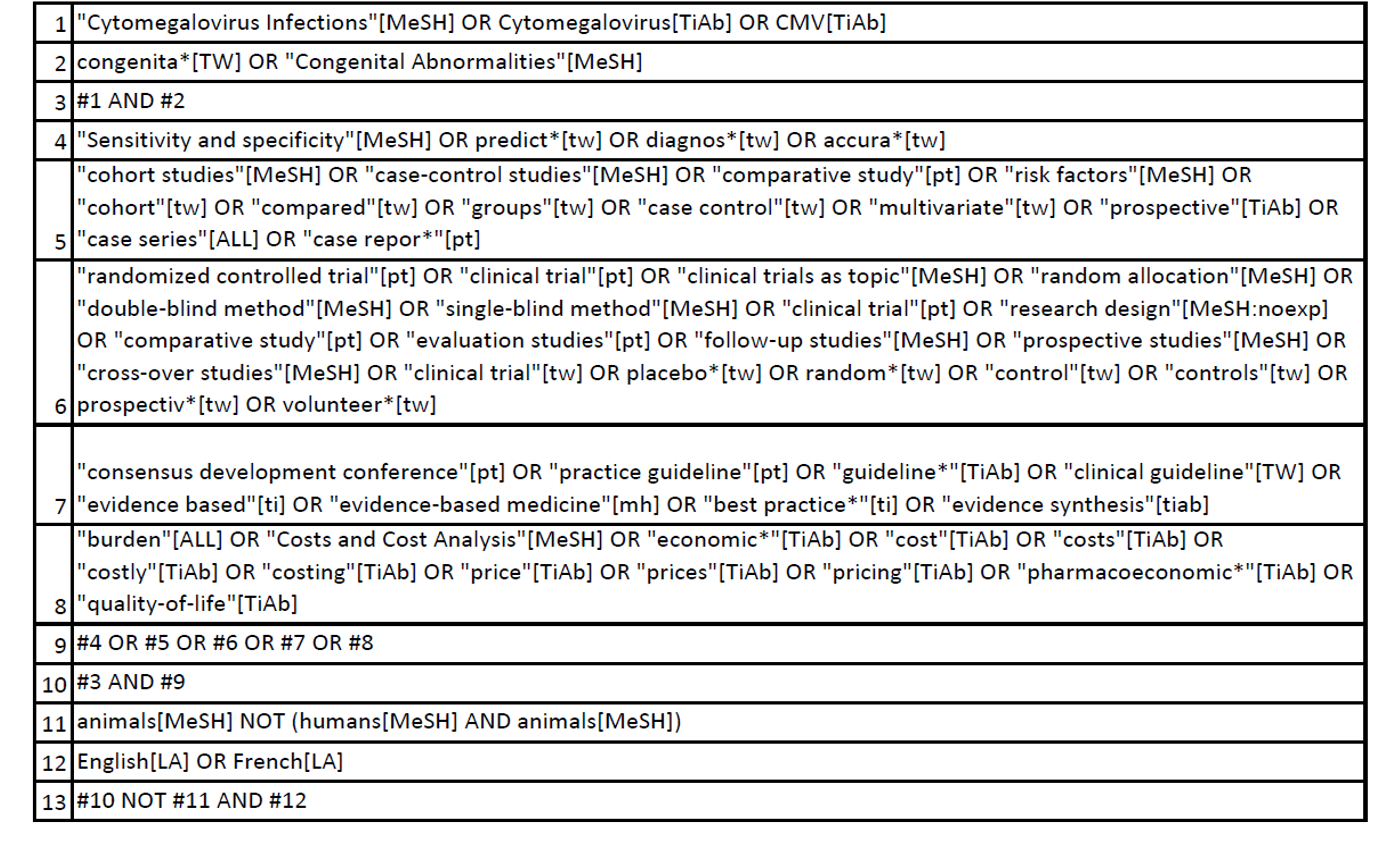

Supplement: S1 Fig — (TIF) [file pone.0261011.s001.tif]

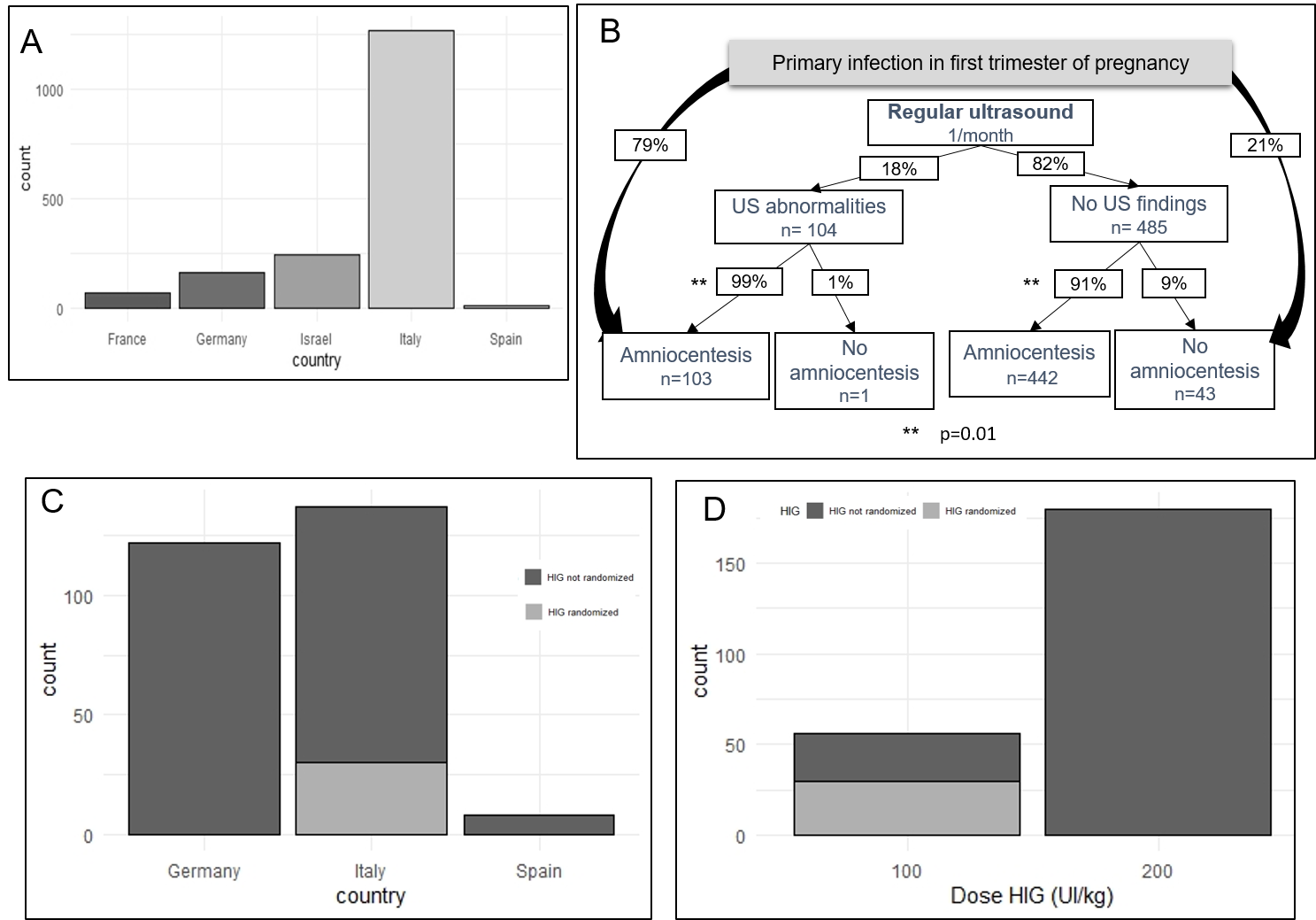

Supplement: S2 Fig — A. Repartition of population 1 according to their country of study. B. Population 1 flowchart with radiological interventions (US and or RMI). Amniocentesis is described according to US abnormalities or not. C. Repartition of patients receiving HIG for preventive treatment according to their country. D. Repartition of patients receiving HIG for preventive treatment according to dose of HIG: 100 UI/kg or 200 UI/kg. Patients receiving HIG in RCT are colored in middle grey and patients receiving HIG in observational studies are colored in dark grey. US: ultrasound; HIG: hyperimmunoglobulin; RCT: randomized controlled trial. (TIF) [file pone.0261011.s002.tif]

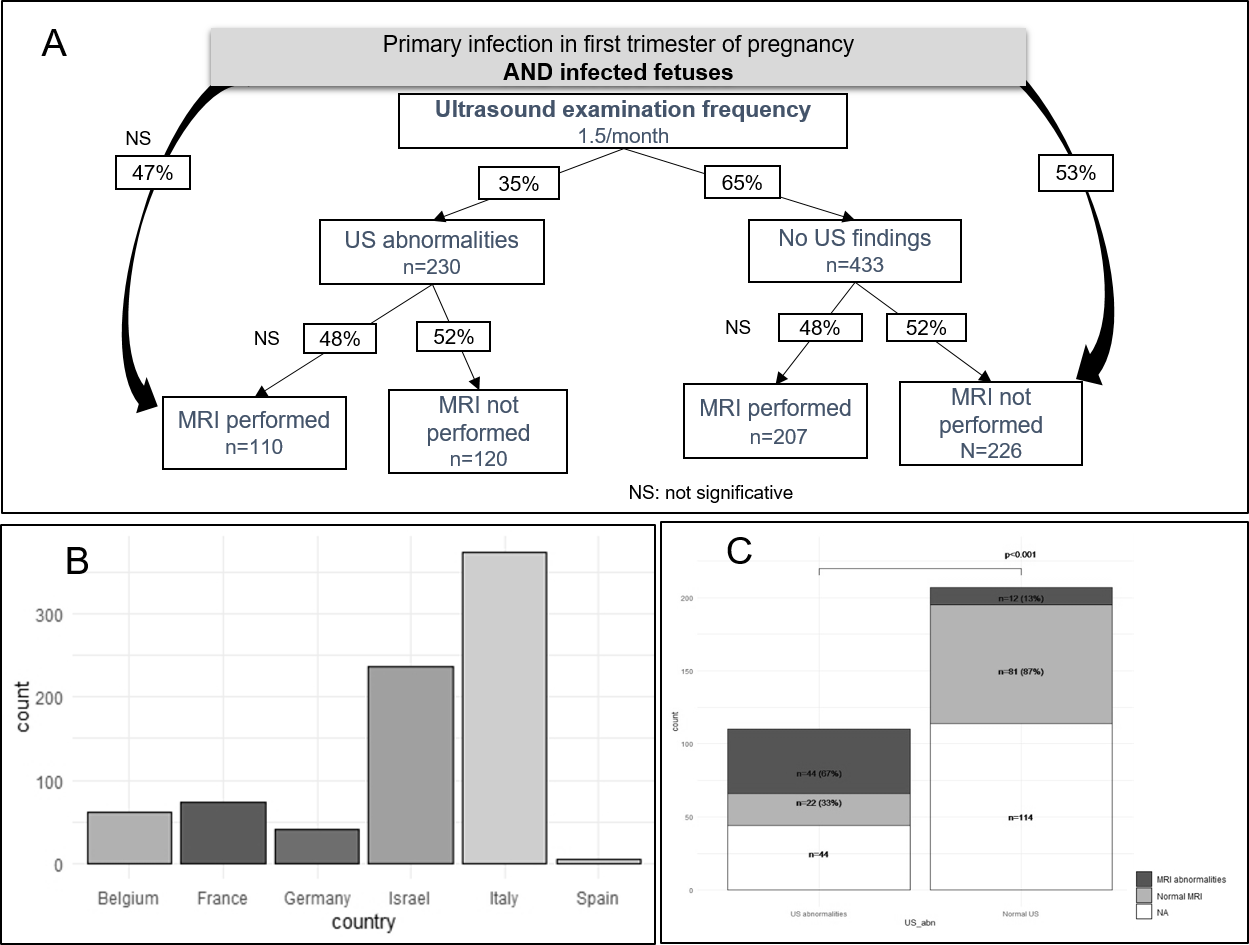

Supplement: S3 Fig — A. Population 2 flowchart with radiological interventions (US and MRI). MRI performing is described according to US abnormalities or not. B. Repartition of population 2 according to their country of study. C. MRI findings according to US abnormalities. NA: data not reported; US: ultrasound; MRI: magnetic resonance imaging. (TIF) [file pone.0261011.s003.tif]

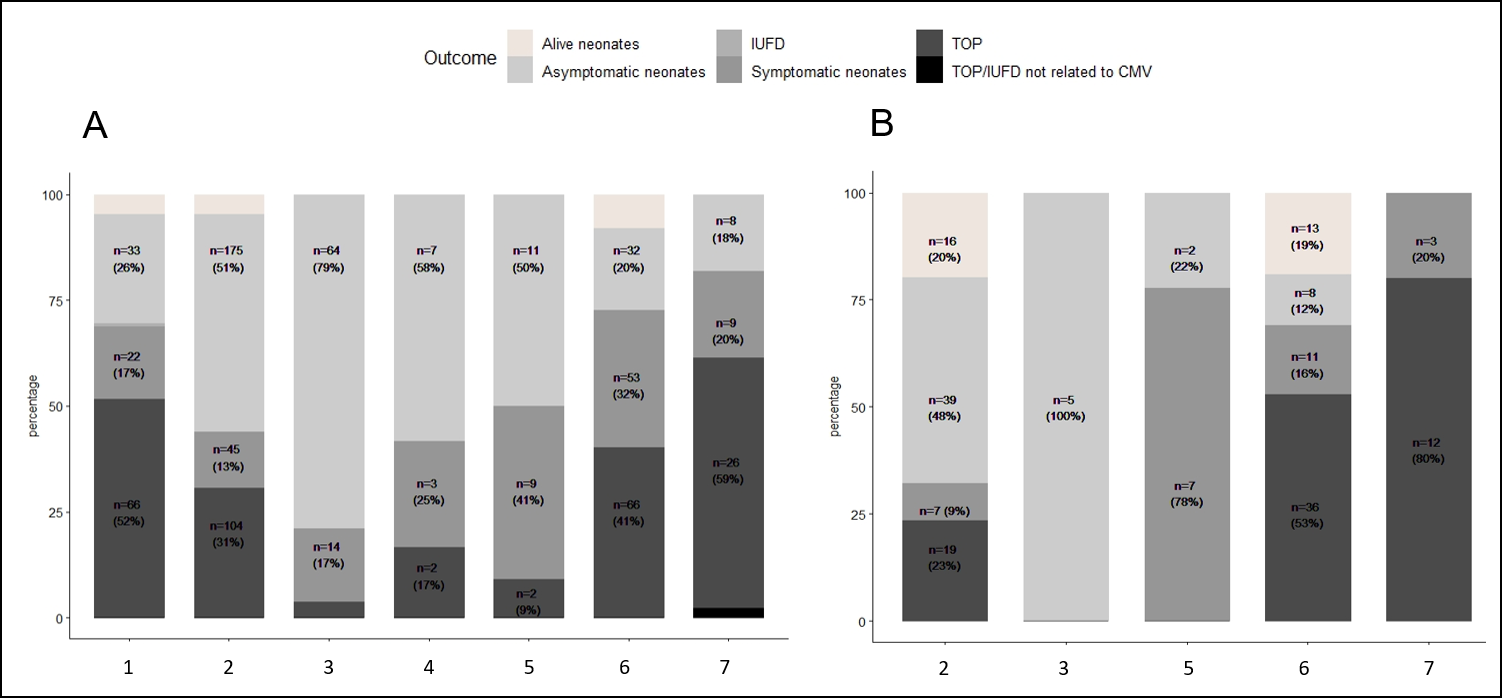

Supplement: S4 Fig — Pregnancy outcome (alive neonate–symptoms not reported, asymptomatic neonate, IUFD, symptomatic neonate, TOP, TOP/IUFD not related to cCMV) is reported according to radiological findings (1 to 7)) in sensitivity analysis for population with infected fetuses. A. First sensitivity analysis: without studies with fair risk of bias B. Second sensitivity analysis: without studies with fair risk of bias and without studies with nor Italian patients nor Israeli patients. 1: presence of US findings? Data not reported; 2: Normal US; 3: Normal US and normal MRI; 4: Normal US and MRI abnormalities; 5: US abnormalities and normal MRI; 6: US abnormalities; 7: US abnormalities and MRI abnormalities. US: ultrasound; MRI: magnetic resonance imaging; TOP: termination of pregnancy; IUFD: intra uterine fetal death. (TIF) [file pone.0261011.s004.tif]
