# Supplementary material for: Current practices of management of maternal and congenital Cytomegalovirus infection during pregnancy after a maternal primary infection occurring in first trimester of pregnancy: Systematic review
Source: PLoS One. 2021 Dec 3;16(12):e0261011. doi: 10.1371/journal.pone.0261011 (PMC8641894; doi:10.1371/journal.pone.0261011)
Supplement: S1 Table — (DOCX) [file pone.0261011.s005.docx]

**S1 Table**: Description of excluded studies on full text and reasons of exclusion

| Author | PICOS reason of exclusion | Reason of exclusion |
| --- | --- | --- |
| Sert *et al.* (1) | I | Lack of description of interventions |
| Yefet E. et al. (2) | I | Lack of description of interventions |
| Rymer-Haskel N *et al.* (3) | I | Lack of description of interventions |
| Toriyabe K *et al.* (4) | I | Not interventions of interest |
| McCrary H *et al.* (5) | P | Not population of interest |
| Faure-Bardon, Valentine *et al.* (6) | P | Not population of interest |
| Albright, Catherine M *et al.* (7) | I | Not interventions of interest |
| Marsico, Concetta *et al.* (8) | P | Not population of interest |
| Barlinn, Regine *et al.* (9) | I | Not interventions of interest |
| Penka, Lukas *et al.* (10) | I | Not interventions of interest |
| Zavattoni, Maurizio *et al.* (11) | P | Description of management for less than 10 children |
| Bartlett, Adam W *et al.* (12) | P | Not population of interest |
| Minsart, Anne-Frédérique *et al.* (13) | P | Lack of description of population and interventions |
| Mussi-Pinhata, Marisa M *et al.* (14) | P | Not population of interest |
| Delforge, Marie-Luce *et al.* (15) | I | Not interventions of interest |
| Kaneko, Masatoki *et al.* (16) | I | Not interventions of interest |
| Inagaki, Kengo *et al.* (17) | I | Not interventions of interest |
| Furione, Milena *et al.* (18) | P | Less than 10 women with first trimester primary infection |
| Korndewal, Marjolein J *et al.* (19) | I | Not interventions of interest |
| Puhakka, Laura *et al.* (20) | P | Less than 10 women with first trimester primary infection |
| Isik, Dilek Ulubas *et al.* (21) | P | Less than 10 women with first trimester primary infection |
| Tshabalala, Donald *et al.* (22) | P | Not population of interest |
| Yao H.T. (23) | P | Less than 10 women with first trimester primary infection |
| Toriyabe, K *et al.* (24) | I | Not interventions of interest |
| Tanimura, Kenji *et al.* (25) | I | Not interventions of interest |
| Kotovich, D *et al.* (26) | I | Not interventions of interest |
| Tanimura, Kenji *et al.* (27) | P | Not population of interest |
| Tairaku, Shinya *et al.* (28) | P | Less than 10 women with first trimester primary infection |
| Kollarova, K *et al.* (29) | I | Not interventions of interest |
| Mujtaba, Ghulam *et al.* (30) | I | Not interventions of interest |
| Lin, Xiaoqian *et al.* (31) | P | Less than 10 women with first trimester primary infection |
| Carrara, J *et al.* (32) | P | Not population of interest |
| Zavattoni, Maurizio *et al.* (33) | I | Lack of description of interventions |
| Carrara, Julie *et al.* (34) | P | Not population of interest |
| De La Calle M *et al.* (35) | overlap | Overlap with Blazquez-Gamero 2019 |
| Terhes G *et al.* (36) | I | Not interventions of interest |
| Shand, Antonia W. *et al.* (37) | S | Not study design of interest |
| Yan, Xiao Chuan *et al.* (38) | I | Not interventions of interest |
| Kobayashi, Yoko *et al.* (39) | I | Not interventions of interest |
| Nigro, Giovanni *et al.* (40) | P | Lack of description of population |
| Blázquez-Gamero D *et al*. (41) | Overlap | Overlap with Blazquez-Gamero 2019 |
| Ebina, Yasuhiko *et al*. (42) | I | Not interventions of interest |
| Tanimura, Kenji *et al*. (43) | P | Less than 10 women with first trimester primary infection |
| Picone, O *et al*. (44) | P | Lack of description of population |
| Vergani P *et al*. (45) | S | Congress abstract > 3 years |
| NCT01037712 (46) | Overlap | Overlap with Leruez-Ville 2016 |
| Picone, Olivier *et al*. (47) | P | Lack of description of population |
| Alarcon, Ana *et al*. (48) | P | Not population of interest |
| Pass, Robert F *et al*. (49) | I | Lack of description of interventions |
| Chakravarty, Anita *et al*. (50) | I | Not interventions of interest |
| de Vries, L. S. *et al*. (51) | I | Lack of description of interventions |
| Lehman, D *et al*. (52) | S | Congress abstract > 3 years |
| Guerra, B *et al*. (53) | Overlap | Overlap with Guerra 2008 |
| Revello, M. G *et al*. (54) | P | Less than 10 women with first trimester primary infection |
| Lumley, S *et al*. (55) | P | Less than 10 women with first trimester primary infection |
| Teissier, Natacha *et al*. (56) | I | Not interventions of interest |
| Fernández-Zubillaga A *et al*. (57) | S | Congress abstract > 3 years |
| De La Calle M *et al*. (58) | Overlap | Overlap with Blazquez-Gamero 2019 |
| Leruez-Ville, Marianne *et al*. (59) | Overlap | Overlap with Leruez-Ville 2016 |
| Yoshida, Masashi *et al*. (60) | P | Lack of description of population |
| Yamada H. (61) | S | Congress abstract > 3 years |
| NCT02005822 (62) | P | Not population of interest |
| Tairaku S *et al*. (63) | S | Congress abstract > 3 years |
| Lazzarotto T. (64) | I | Lack of description of interventions |
| De La Calle M *et al*. (65) | S | Congress abstract > 3 years |
| Sonoyama, Ayako *et al*. (66) | P | Not population of interest |
| Nigro, Giovanni *et al*. (67) | P | Not population of interest |
| Japanese Congenital Cytomegalovirus Infection Immunoglobulin Fetal Therapy Study Group (68) | I | Lack of description of interventions |
| Gabbay-Benziv, Rinat *et al*. (69) | P | Less than 10 women with first trimester primary infection |
| Goncé, Anna *et al*. (70) | I | Not interventions of interest |
| del Rosal, Teresa *et al.* (71) | P | Not population of interest |
| Buxmann, Horst *et al*. (72) | Overlap | Overlap with Chiaie 2018 |
| Levitt, Lorinne *et al*. (73) | S | Congress abstract > 3 years |
| Furione M et al. (74) | S | Congress abstract > 3 years |
| Polilli, E et al. (75) | P | Not population of interest |
| Enders, Gisela *et al*. (76) | I | Not intervention of interest |
| Farkas, Natalie *et al*. (77) | P | Lack of description of population |
| Fabbri, E *et al*. (78) | P | Lack of description of population |
| Buxmann H *et al*. (79) | S | Congress abstract > 3 years |
| Doneda, Chiara *et al*. (80) | P | Lack of description of population |
| Kabartas I.B *et al*. (81) | S | Congress abstract > 3 years |
| Gatta E *et al*. (82) | S | Congress abstract > 3 years |
| Goncé A *et al*. (83) | S | Congress abstract > 3 years |
| Lazzarotto T *et al*. (84) | S | Congress abstract > 3 years |
| Puccetti C *et al*. (85) | S | Congress abstract > 3 years |
| Faure-Bardon *et al.* (86) | I | Not intervention of interest |
| Tanimura *et al.*(87) | P | Not population of interest |
| De La Calle *et al.* (88) | P | Less than 10 women with first trimester primary infection |
| Belooseski *et al*. (89) |  | High risk of bias |
| Cannie *et al.* (90) |  | High risk of bias |
| Enders *et al*. (91) | Overlap | High risk of overlap with Enders 2017 |

**References S1 Table**

1. Sert Y, Ozgu-Erdinc AS, Saygan S, Engin Ustun Y. Antenatal Cytomegalovirus Infection Screening Results of 32,188 Patients in a Tertiary Referral Center: A Retrospective Cohort Study. Fetal Pediatr Pathol. 2019 Jan 2;1–9.

2. Yefet E., Ben Shmuel Y., Nachum Z. 631: The association between polyhydramnios and CMV infection. Am J Obstet Gynecol. 2019;220(1):S417–8.

3. Rymer-Haskel N., Barkai G., Duvdevani N., Weisz B., Lipitz S., Yinon Y. 651: Neurological outcome of congenital cytomegalovirus infection following primary maternal infection and a negative amniocentesis. Am J Obstet Gynecol. 2019;220(1):S432.

4. Toriyabe K., Shimada K., Kitamura A., Minematsu T., Ikejiri M., Suga S., et al. 8: Primary cytomegalovirus infection during pregnancy and subsequent congenital infection: maternal antibody screening involving 19,000 women. Am J Obstet Gynecol. 2019;220(1):S6–7.

5. McCrary H., Sheng X., Greene T., Park A. Long-term hearing outcomes of children with symptomatic congenital CMV treated with valganciclovir. Int J Pediatr Otorhinolaryngol. 2019;118((McCrary H.; Park A., albert.park@hsc.utah.edu) University of Utah School of Medicine, Division of Otolaryngology-Head and Neck Surgery, United States):124–7.

6. Faure-Bardon V, Magny J-F, Parodi M, Couderc S, Garcia P, Maillotte A-M, et al. Sequelae of congenital cytomegalovirus (cCMV) following maternal primary infection are limited to those acquired in the first trimester of pregnancy. Clin Infect Dis Off Publ Infect Dis Soc Am. 2018 Dec 31;

7. Albright CM, Werner EF, Hughes BL. Cytomegalovirus Screening in Pregnancy: A Cost-Effectiveness and Threshold Analysis. Am J Perinatol. 2018 Dec 19;

8. Marsico C, Aban I, Kuo H, James SH, Sanchez PJ, Ahmed A, et al. Blood Viral Load in Symptomatic Congenital Cytomegalovirus Infection. J Infect Dis. 2018 Dec 7;

9. Barlinn R, Dudman SG, Trogstad L, Gibory M, Muller F, Magnus P, et al. Maternal and congenital cytomegalovirus infections in a population-based pregnancy cohort study. APMIS Acta Pathol Microbiol Immunol Scand. 2018 Dec;126(12):899–906.

10. Penka L, Kagan K-O, Goelz R, Hamprecht K. Comparison of quantitative real-time PCR and short-term (18-hour) microculture in diagnosis of fetal cytomegalovirus infection: Impact of hyperimmunoglobulin treatment. Prenat Diagn. 2018 Nov;38(12):936–42.

11. Zavattoni M, Lombardi G, Garofoli F, Scalia G, Rizzo A, Angelini M, et al. Neonatal HCMV-related polymicrogyria in seroimmune women: What is the optimal pregnancy management? J Clin Virol. 2018 Nov;108:141–6.

12. Bartlett AW, Hall BM, Palasanthiran P, McMullan B, Shand AW, Rawlinson WD. Recognition, treatment, and sequelae of congenital cytomegalovirus in Australia: An observational study. J Clin Virol Off Publ Pan Am Soc Clin Virol. 2018 Nov;108:121–5.

13. Minsart A-F, Smiljkovic M, Renaud C, Gagné M-P, Lamarre V, Kakkar F, et al. Use of Cytomegalovirus-Specific Hyperimmunoglobulins in Pregnancy: A Retrospective Cohort. J Obstet Gynaecol Can JOGC J Obstet Gynecol Can JOGC. 2018 Nov;40(11):1409–16.

14. Mussi-Pinhata MM, Yamamoto AY, Aragon DC, Duarte G, Fowler KB, Boppana S, et al. Seroconversion for Cytomegalovirus Infection During Pregnancy and Fetal Infection in a Highly Seropositive Population: ‘The BraCHS Study’. J Infect Dis. 2018 Sep 8;218(8):1200–4.

15. Delforge M-L, Costa E, Brancart F, Goldman D, Montesinos I, Zaytouni S, et al. Presence of Cytomegalovirus in urine and blood of pregnant women with primary infection might be associated with fetal infection. J Clin Virol. 2017 May;90:14–7.

16. Kaneko M, Sameshima H, Minematsu T, Kusumoto K, Yamauchi A, Ikenoue T. Maternal IgG avidity, IgM and ultrasound abnormalities: combined method to detect congenital cytomegalovirus infection with sequelae. J Perinatol Off J Calif Perinat Assoc. 2013 Nov;33(11):831–5.

17. Inagaki K, Blackshear C, Palmer A, Hobbs CV. Risk Factors, Geographic Distribution, and Healthcare Burden of Symptomatic Congenital Cytomegalovirus Infection in the United States: Analysis of a Nationally Representative Database, 2000-2012. J Pediatr. 2018 Aug;199:118-123.e1.

18. Furione M, Sarasini A, Arossa A, Fornara C, Lilleri D, Perez L, et al. False human cytomegalovirus IgG-positivity at prenatal screening. J Clin Virol Off Publ Pan Am Soc Clin Virol. 2018 Jul;104:34–8.

19. Korndewal MJ, Weltevrede M, van den Akker-van Marle ME, Oudesluys-Murphy AM, de Melker HE, Vossen ACTM. Healthcare costs attributable to congenital cytomegalovirus infection. Arch Dis Child. 2018 May;103(5):452–7.

20. Puhakka L, Lappalainen M, Lönnqvist T, Niemensivu R, Lindahl P, Nieminen T, et al. The Burden of Congenital Cytomegalovirus Infection: A Prospective Cohort Study of 20 000 Infants in Finland. J Pediatr Infect Dis Soc. 2018 Mar 15;

21. Isik DU, Ozcan B, Demirel N, Unal S, Celik IH, Bas AY. Case Series of Congenital Cytomegalovirus Infection in Neonatal Period. Gazi Med J. 2018;29(1):31–3.

22. Tshabalala D, Newman H, Businge C, Mabunda SA, Kemp W, Beja P. Prevalence and determinants of congenital cytomegalovirus infection at a rural South African central hospital in the Eastern Cape. South Afr J Infect Dis. 2018;33(4):89–92.

23. Yao H.T. Sero-epidemiological survey of cytomegalovirus infection among pregnant women in Taiwan. Int J Gynecol Obstet. 2018;143((Yao H.T.) Chang Gung Memorial Hospital, Kaohsiung, Taiwan):708.

24. Toriyabe K., Morikawa F., Minematsu T., Kitamura A., Shimada K., Kubo M., et al. Anticytomegalovirus IgM titer for congenital infection in first-trimester pregnancy with primary infection. J Obstet Gynaecol Res. 2018;44(8):1555–6.

25. Tanimura K, Tairaku S, Morioka I, Ozaki K, Nagamata S, Morizane M, et al. Universal Screening With Use of Immunoglobulin G Avidity for Congenital Cytomegalovirus Infection. Clin Infect Dis Off Publ Infect Dis Soc Am. 2017 Oct 30;65(10):1652–8.

26. Kotovich D, Guedalia JSB, Hoffmann C, Sze G, Eisenkraft A, Yaniv G. Apparent Diffusion Coefficient Value Changes and Clinical Correlation in 90 Cases of Cytomegalovirus-Infected Fetuses with Unremarkable Fetal MRI Results. AJNR Am J Neuroradiol. 2017 Jul;38(7):1443–8.

27. Tanimura K, Tairaku S, Ebina Y. Prediction of congenital cytomegalovirus infection in high-risk pregnant women (vol 64, pg 159, 2017). Clin Infect Dis. 2017 May 15;64(10):1469–1469.

28. Tairaku S, Tanimura K, Morioka I, Ozaki K, Nagamata S, Morizane M, et al. Effectiveness of maternal serological screening for congenital cytomegalovirus infection. J Reprod Immunol. 2016 Nov;118:119–119.

29. Kollarova K, Huckova D. Practical experience in laboratory diagnosis of congenital CMV infection over a period of 8 years in Slovakia. J Clin Virol. 2016 Sep;82:S134–5.

30. Mujtaba G, Shaukat S, Angez M, Alam MM, Hasan F, Zaidi SSZ, et al. Seroprevalence of Human Cytomegalovirus (HCMV) infection in pregnant women and outcomes of pregnancies with active infection. J Pak Med Assoc. 2016 Aug;66(8):1009–14.

31. Lin X, Wang J, Wang Z, Ru T, Dai Y, Li J, et al. Rare detection of cytomegalovirus in severe fetal malformations in China. J Clin Virol. 2016 Jun;79:54–60.

32. Carrara J, N’Diaye DS, Azria E, Launay O, Rozenberg F, Yazpandanah Y, et al. Management of Cytomegalovirus Seroconversion during Pregnancy in France. Fetal Diagn Ther. 2016;39(1):4–12.

33. Zavattoni M, Rustico M, Tassis B, Lombardi G, Furione M, Piralla A, et al. Risk of congenital disease in 46 infected fetuses according to gestational age of primary human cytomegalovirus infection in the mother: Risk of HCMV Congenital Disease. J Med Virol. 2016 Jan;88(1):120–6.

34. Carrara J, Delaveaucoupet J, Cordier AG, Vauloup-Fellous C, Senat MV, Ayoubi JM, et al. Étude descriptive de signes échographiques anténataux de 34 cas d’infections congénitales à cytomégalovirus. J Gynécologie Obstétrique Biol Reprod. 2016 Apr;45(4):397–406.

35. De La Calle M., Blázquez-Gamero D., Galindo Izquierdo A., Baquero-Artigao F., Izquierdo Méndez N., Soriano-Ramos M., et al. Prevention and treatment of fetal cytomegalovirus infection with CMV-hyperimmune globulin: A multicentre study in Madrid (GECITMA Group). J Matern Fetal Neonatal Med. 2016;29((Blázquez-Gamero D.; Soriano-Ramos M.; Rojo Conejo P.; González-Tomé M.I.; Sánchez V.) Pediatric Infectious Disease Unit, Hospital Universitario 12 De Octubre, Spain):30–1.

36. Terhes G., Szcs M., Pál Z., Németh G., Urbán E. Screening for cytomegalovirus infection in pregnancy, early findings of a single-center prospective survey. Clin Chem Lab Med. 2016;54(10):eA178.

37. Shand AW. The obstetrician, congenital cytomegalovirus, clinical and diagnostic approaches to the pregnant woman. Microbiol Aust. 2015 Nov;36(4):194–6.

38. Yan XC, Wang JH, Wang B, Huang LL, Zhou LQ, Zhu B, et al. Study of Human Cytomegalovirus Replication in Body Fluids, Placental Infection, and Miscarriage During the First Trimester of Pregnancy. J Med Virol. 2015 Jun;87(6):1046–53.

39. Kobayashi Y, Morioka I, Koda T, Nakamachi Y, Okazaki Y, Noguchi Y, et al. Low total IgM values and high cytomegalovirus loads in the blood of newborns with symptomatic congenital cytomegalovirus infection. J Perinat Med. 2015 Mar;43(2):239–43.

40. Nigro G, Capretti I, Manganello A-M, Best AM, Adler SP. Primary maternal cytomegalovirus infections during pregnancy: association of CMV hyperimmune globulin with gestational age at birth and birth weight. J Matern-Fetal Neonatal Med Off J Eur Assoc Perinat Med Fed Asia Ocean Perinat Soc Int Soc Perinat Obstet. 2015 Jan;28(2):168–71.

41. Blázquez-Gamero D., De La Calle M., Galindo Izqueirdo A., Baquero-Artigao F., Izquierdo Méndez N., Soriano-Ramos M., et al. Treatment of fetal cytomegalovirus infection with CMV-hyperimmune globulin. J Perinat Med [Internet]. 2015;43((Blázquez-Gamero D.; Galindo Izqueirdo A.; Soriano-Ramos M.; Rojo P.; González-Tomé M.I.; García-Burguillo A.; Sánchez V.) Hospital 12 De Octubre, Madrid, Spain). Available from: http://www.embase.com/search/results?subaction=viewrecord&from=export&id=L72184990

42. Ebina Y, Minematsu T, Sonoyama A, Morioka I, Inoue N, Tairaku S, et al. The IgG avidity value for the prediction of congenital cytomegalovirus infection in a prospective cohort study. J Perinat Med. 2014 Nov;42(6):755–9.

43. Tanimura K, Tairaku S, Deguchi M, Sonoyama A, Morizane M, Ebina Y, et al. Prophylactic intravenous immunoglobulin injections to mothers with primary cytomegalovirus infection. Kobe J Med Sci. 2014 Jul 11;60(2):E25-29.

44. Picone O, Teissier N, Cordier AG, Vauloup-Fellous C, Adle-Biassette H, Martinovic J, et al. Detailed in utero ultrasound description of 30 cases of congenital cytomegalovirus infection: Detailed in utero ultrasound description of congenital cytomegalovirus infection. Prenat Diagn. 2014 Jun;34(6):518–24.

45. Vergani P., Ornaghi S., Verderio M., Ceruti P., Follesa I., Russo F.M., et al. Cytomegalovirus infection in pregnancy: Role of serial ultrasounds. Am J Obstet Gynecol. 2009;201(6):S145.

46. NCT01037712. In UTERO Treatment of Cytomegalovirus Congenital Infection With Valacyclovir. Https://clinicaltrials.gov/show/nct01037712 [Internet]. 2009; Available from: https://www.cochranelibrary.com/central/doi/10.1002/central/CN-01527339/full

47. Picone O, Simon I, Benachi A, Brunelle F, Sonigo P. Comparison between ultrasound and magnetic resonance imaging in assessment of fetal cytomegalovirus infection. Prenat Diagn. 2008 Aug;28(8):753–8.

48. Alarcon A, Garcia-Alix A, Cabañas F, Hernanz A, Pascual-Salcedo D, Martin-Ancel A, et al. Beta2-microglobulin concentrations in cerebrospinal fluid correlate with neuroimaging findings in newborns with symptomatic congenital cytomegalovirus infection. Eur J Pediatr. 2006 Sep;165(9):636–45.

49. Pass RF, Fowler KB, Boppana SB, Britt WJ, Stagno S. Congenital cytomegalovirus infection following first trimester maternal infection: Symptoms at birth and outcome. J Clin Virol. 2006 Feb;35(2):216–20.

50. Chakravarty A, Kashyap B, Rathi K. The seroepidemiological study on cytomegalovirus in women of child-bearing age with special reference to pregnancy and maternal-fetal transmission. Indian J Pathol Microbiol. 2005 Oct;48(4):518–21.

51. de Vries LS, Gunardi H, Barth PG, Bok LA, Verboon-Maciolek MA, Groenendaal F. The spectrum of cranial ultrasound and magnetic resonance imaging abnormalities in congenital cytomegalovirus infection. Neuropediatrics. 2004 Apr;35(2):113–9.

52. Lehman D, Toyoda M, Jordan S, Silverman NS. Prenatal diagnosis of congenital cytomegalovirus infection: Quantitative CMV PCR in amniotic fluid. Clin Infect Dis. 2001 Oct 1;33(7):1140–1140.

53. Guerra B, Lazzarotto T, Quarta S, Lanari M, Bovicelli L, Nicolosi A, et al. Prenatal diagnosis of symptomatic congenital cytomegalovirus infection. Am J Obstet Gynecol. 2000 Aug;183(2):476–82.

54. Revello MG, Baldanti F, Furione M, Sarasini A, Percivalle E, Zavattoni M, et al. Polymerase chain reaction for prenatal diagnosis of congenital human cytomegalovirus infection. J Med Virol. 1995 Dec;47(4):462–6.

55. Lumley S, Patel M, Griffiths PD. The combination of specific IgM antibodies and IgG antibodies of low avidity does not always indicate primary infection with cytomegalovirus. J Med Virol. 2014 May;86(5):834–7.

56. Teissier N, Fallet-Bianco C, Delezoide A-L, Laquerrière A, Marcorelles P, Khung-Savatovsky S, et al. Cytomegalovirus-induced brain malformations in fetuses. J Neuropathol Exp Neurol. 2014 Feb;73(2):143–58.

57. Fernández-Zubillaga A., Royo A., Utrilla C., De La Calle M., Rodríguez R., Garzón G. The value of prenatal brain MRI in the management of congenital cytomegalovirus infection. Neuroradiology. 2014;56((Fernández-Zubillaga A.; Royo A.; Utrilla C.; Garzón G.) Hospital Universitario la Paz Radiology, Madrid, Spain):194.

58. De La Calle M., Rodriguez R., Baquero F., Fernandez A., Luis Bartha J. Use of cytomegalovirus hyperimmunoglobulin for prevention of congenital cytomegalovirus disease. Am J Obstet Gynecol. 2014;210(1):S215–6.

59. Leruez-Ville M, Sellier Y, Salomon LJ, Stirnemann JJ, Jacquemard F, Ville Y. Prediction of Fetal Infection in Cases With Cytomegalovirus Immunoglobulin M in the First Trimester of Pregnancy: A Retrospective Cohort. Clin Infect Dis. 2013 May 15;56(10):1428–35.

60. Yoshida M, Matsuda H, Yoshinaga Y, Asai K, Kawashima A, Sei K, et al. Can measurement of maternal anti-cytomegalovirus immunoglobulin-M antibody levels be used to screen for cytomegalovirus infection in embryos and fetuses? J Obstet Gynaecol Res. 2013 Jan;39(1):166–9.

61. Yamada H. A trial of immunoglobulin fetal therapy for symptomatic congenital cytomegalovirus infection. Prenat Diagn. 2013;33((Yamada H.) Department of Obstetrics and Gynecology, Kobe University, Graduate School of Medicine, Kobe, Japan):66–7.

62. NCT02005822. Congenital Cytomegalovirus: efficacy of Antiviral Treatment. Https://clinicaltrials.gov/show/nct02005822 [Internet]. 2013; Available from: https://www.cochranelibrary.com/central/doi/10.1002/central/CN-01479595/full

63. Tairaku S., Sonoyama A., Ebina Y., Morioka I., Tanimura K., Minematsu T., et al. Low IgG avidity and ultrasound fetal abnormality predict congenital cytomegalovirus infection. Prenat Diagn. 2013;33((Tairaku S.; Sonoyama A.; Ebina Y.; Tanimura K.; Yamada H.) Department of Obstetrics and Gynecology, Kobe University, Graduate School of Medicine, Kobe, Hyogo, Japan):63.

64. Lazzarotto T. Pre-and post-natal diagnosis of congenital cytomegalovirus infection. Biochim Clin. 2013;37((Lazzarotto T.) Operative Unit of Clinical Microbiology, General University Hospital S. Orsola-Malpighi, University of Bologna, Italy):S55.

65. De La Calle M., Herrero B., Baquero F., Fernandez A., Omeñaca F., Bartha J.L. Use of cytomegalovirus hyperimmunoglobulin during pregnancy for prevention of congenital cytomegalovirus disease. J Perinat Med [Internet]. 2013;41((De La Calle M.; Herrero B.; Bartha J.L.) Obstetrics Department, United States). Available from: http://www.embase.com/search/results?subaction=viewrecord&from=export&id=L71115054

66. Sonoyama A, Ebina Y, Morioka I, Tanimura K, Morizane M, Tairaku S, et al. Low IgG avidity and ultrasound fetal abnormality predict congenital cytomegalovirus infection. J Med Virol. 2012 Dec;84(12):1928–33.

67. Nigro G, Adler SP, Gatta E, Mascaretti G, Megaloikonomou A, La Torre R, et al. Fetal hyperechogenic bowel may indicate congenital cytomegalovirus disease responsive to immunoglobulin therapy. J Matern-Fetal Neonatal Med Off J Eur Assoc Perinat Med Fed Asia Ocean Perinat Soc Int Soc Perinat Obstet. 2012 Nov;25(11):2202–5.

68. Japanese Congenital Cytomegalovirus Infection Immunoglobulin Fetal Therapy Study Group. A trial of immunoglobulin fetal therapy for symptomatic congenital cytomegalovirus infection. J Reprod Immunol. 2012 Sep;95(1–2):73–9.

69. Gabbay-Benziv R, Gabbay-Ben Ziv R, Yogev Y, Peled Y, Amir J, Pardo J. Congenital cytomegalovirus infection following antenatal negative diagnostic amniotic fluid analysis - a single center experience. J Matern-Fetal Neonatal Med Off J Eur Assoc Perinat Med Fed Asia Ocean Perinat Soc Int Soc Perinat Obstet. 2012 Sep;25(9):1787–90.

70. Goncé A, Marcos MA, Borrell A, López M, Nadal A, Figueras F, et al. Maternal IgM antibody status in confirmed fetal cytomegalovirus infection detected by sonographic signs. Prenat Diagn. 2012 Sep;32(9):817–21.

71. del Rosal T, Baquero-Artigao F, Blázquez D, Noguera-Julian A, Moreno-Pérez D, Reyes A, et al. Treatment of symptomatic congenital cytomegalovirus infection beyond the neonatal period. J Clin Virol Off Publ Pan Am Soc Clin Virol. 2012 Sep;55(1):72–4.

72. Buxmann H, Stackelberg OM v, Schlößer RL, Enders G, Gonser M, Meyer-Wittkopf M, et al. Use of cytomegalovirus hyperimmunoglobulin for prevention of congenital cytomegalovirus disease: a retrospective analysis. J Perinat Med. 2012 Mar 27;40(4):439–46.

73. Levitt L., Valsky D.V., Yanai N., Hochner-Celnikier D., Wolf D., Yagel S. Congenital CMV infection: Limits of viral load and prenatal imaging in predicting infection and disease during mid and late pregnancy. Reprod Sci. 2012;19(3):95A.

74. Furione M., Zavattoni M., Rognoni V., Arossa A., Nazifi M., Formica M., et al. Insights into human cytomegalovirus infection in utero following detection of low viral DNA amounts in amniotic fluid. J Matern Fetal Neonatal Med. 2012;25((Furione M.; Zavattoni M.; Rognoni V.) SC Virologia e Microbiologia, IRCCS Policlinico, San Matteo, Pavia, Italy):28–9.

75. Polilli E, Parruti G, D’Arcangelo F, Tracanna E, Clerico L, Savini V, et al. Preliminary evaluation of the safety and efficacy of standard intravenous immunoglobulins in pregnant women with primary cytomegalovirus infection. Clin Vaccine Immunol. 2012;19(12):1991‐1993.

76. Enders G, Daiminger A, Bäder U, Exler S, Enders M. Intrauterine transmission and clinical outcome of 248 pregnancies with primary cytomegalovirus infection in relation to gestational age. J Clin Virol. 2011 Nov;52(3):244–6.

77. Farkas N, Hoffmann C, Ben-Sira L, Lev D, Schweiger A, Kidron D, et al. Does normal fetal brain ultrasound predict normal neurodevelopmental outcome in congenital cytomegalovirus infection? Prenat Diagn. 2011 Apr;31(4):360–6.

78. Fabbri E, Revello MG, Furione M, Zavattoni M, Lilleri D, Tassis B, et al. Prognostic markers of symptomatic congenital human cytomegalovirus infection in fetal blood. BJOG Int J Obstet Gynaecol. 2011 Mar;118(4):448–56.

79. Buxmann H., Von Stackelberg O.M., Schloesser R.L., Enders G., Gonser M., Meyer-Wittkopf M., et al. Cytomegalovirus hyperimmune globuline (HIG) for pregnant women to prevent congenital CMV-disease: Retrospective results from 42 mother-child Pairs. Intensive Care Med. 2011;37((Buxmann H.; Von Stackelberg O.M.; Schloesser R.L.) Neonatology, University Children’s Hospital, Frankfurt/Main, Germany):S353.

80. Doneda C, Parazzini C, Righini A, Rustico M, Tassis B, Fabbri E, et al. Early cerebral lesions in cytomegalovirus infection: prenatal MR imaging. Radiology. 2010 May;255(2):613–21.

81. Kabartas I.B., Stinshoff V.J., Chow O., Larbig A., Gonser M. Effect of passive CMV-isoimmunization in pregnancies with seroconversion. Arch Gynecol Obstet. 2010;282((Kabartas I.B.; Stinshoff V.J.; Chow O.; Larbig A.; Gonser M.) HSK-Wiesbaden, Department of Obstetrics and Prenatal Medicine, Wiesbaden, Germany):S77.

82. Gatta E., Anceschi M., Di Renzo G.C., Carrera F., Gussetti N., Mascaretti G., et al. Hyperimmunoglobulin therapy for early or symptomatic fetal cytomegalovirus infection. J Matern Fetal Neonatal Med. 2010;23((Gatta E.; Anceschi M.; Di Renzo G.C.; Carrera F.; Gussetti N.; Mascaretti G.; Pezone I.; Coclite E.; Celli S.; Megaloikonomou A.; Mattia E.; Carta G.; Clerici F.; Nigro G.) Pediatric, L’Aquila’ S University, Italy):362.

83. Goncé A., López M., Angeles Marcos M.A., Puerto Bi., Coll O., Gratacós E. Maternal serological status at the time of ultrasound suspicion in fetuses with confirmed cytomegalovirus infection. J Matern Fetal Neonatal Med. 2010;23((Goncé A.; López M.; Angeles Marcos M.A.; Puerto Bi.; Coll O.; Gratacós E.) Institut Clínic de Ginecologia, Obstetrícia i Neonatologia. Hospital Clínic, Maternal-Fetal Medicine department, Spain):440.

84. Lazzarotto T., Gabrielli L., Baccolini F., Guerra B., Puccetti C., Lanari M., et al. Prenatal diagnosis of congenital cytomegalovirus infection and outcome in 598 pregnant women undergoing a primary cytomegalovirus infection. Clin Microbiol Infect. 2010;16((Lazzarotto T.; Gabrielli L.; Baccolini F.; Guerra B.; Puccetti C.; Lanari M.; Chiereghin A.; Petrisli E.; Piccirilli G.; Landini M.P.) Bologna, Italy):S326.

85. Puccetti C., Guerra B., Cervi F., Vagnoni S., Lazzarotto T., Gabrielli L., et al. Cytomegalovirus DNA load in amniotic fluid and neonatal outcome. Am J Obstet Gynecol. 2009;201(6):S41.

86. Faure-Bardon V, Fourgeaud J, Guilleminot T, Magny J-F, Salomon LJ, Bernard J-P, et al. First-trimester diagnosis of congenital cytomegalovirus infection after maternal primary infection in early pregnancy: feasibility study of viral genome amplification by PCR on chorionic villi obtained by CVS. Ultrasound Obstet Gynecol Off J Int Soc Ultrasound Obstet Gynecol. 2021 Apr;57(4):568–72.

87. Tanimura K, Shi Y, Uchida A, Uenaka M, Imafuku H, Ikuta T, et al. Immunoglobulin fetal therapy and neonatal therapy with antiviral drugs improve neurological outcome of infants with symptomatic congenital cytomegalovirus infection. J Reprod Immunol. 2021 Feb;143:103263.

88. De la Calle M, Baquero-Artigao F, Rodríguez-Molino P, Cabanes M, Cabrera M, Antolin E, et al. Combined treatment with immunoglobulin and valaciclovir in pregnant women with cytomegalovirus infection and high risk of symptomatic fetal disease. J Matern-Fetal Neonatal Med Off J Eur Assoc Perinat Med Fed Asia Ocean Perinat Soc Int Soc Perinat Obstet. 2020 Sep 15;1–5.

89. Beloosesky R, Feldblum I, Shrim A, Kertes J, Segal J, Bachar R, et al. Trends in Continuity of Pregnancy in Women with Positive Cytomegalovirus IgM during the First Trimester, 2008-2009. Isr Med Assoc J. 2017 Aug;19(8):484–8.

90. Cannie MM, Devlieger R, Leyder M, Claus F, Leus A, De Catte L, et al. Congenital cytomegalovirus infection: contribution and best timing of prenatal MR imaging. Eur Radiol. 2016 Oct;26(10):3760–9.

91. Enders M, Daiminger A, Exler S, Enders G. Amniocentesis for prenatal diagnosis of cytomegalovirus infection: challenging the 21 weeks’ threshold. Prenat Diagn. 2017;37(9):940–2.
