# Supplementary material for: Current practices of management of maternal and congenital Cytomegalovirus infection during pregnancy after a maternal primary infection occurring in first trimester of pregnancy: Systematic review
Source: PLoS One. 2021 Dec 3;16(12):e0261011. doi: 10.1371/journal.pone.0261011 (PMC8641894; doi:10.1371/journal.pone.0261011)
Supplement: Attachment — Submitted filename: Response to reviewers 2021-10-23.docx [file pone.0261011.s007.docx]

Response to reviewers

***Reviewer #1****: do you have any reference(s) for the practice and legislation regarding TOP in Israel and Italy? it would be very helpful if you can provide some.
Please pay attention to the references. some are incompletely provided, i. e; 14, 16, 21, 22, 23... etc.*

We thank the first reviewer for his interesting suggestion. We added two legislation references (**line 361**):

61: Italian legislation concerning termination of pregnancy (articles 6 and 7): Leggi Normativa: Norme per la tutela sociale della maternita' e sull'interruzione volontaria della gravidanza. (G.U. Serie Pregressa, n. 140 del 22 maggio 1978) Articles 6,7. Available at https://www.altalex.com/documents/leggi/2008/05/09/tutela-sociale-della-maternita-ed-interruzione-volontaria-della-gravidanza

62: Israeli legislation concerning late termination of pregnancy: Ministry of Health Director Circular 23/07 from 19.12.2007 regarding late termination of pregnancy committees. Available at ttps://www.kolzchut.org.il/en/Termination_of_Pregnancy

As required, we completed references that were incomplete.

***Reviewer #2****: The article has covered published data from seven countries. The data compiled is from different studies published in these countries till dates. The statistical analysis is remarkable. The title "Current practices of management of maternal and congenital Cytomegalovirus infection during pregnancy after a maternal primary infection occurring in first trimester of pregnancy: systematic review: The current review included 31 out of 4134 studies after excluding large number of studies. The objective was to extract information about biological. Clinical, imaging and therapeutic practices. However, looking into the data it appears that majority of data is from Italy followed by France. The review could not provide any conclusive new information that can be adopted by a practitioner. The data is variable and scattered. It does not specifically arrive at any specific conclusion about its general objectives.*

We are grateful for the second reviewer for careful reading of our manuscript. Indeed, majority of data is from Italy, followed by France and Israel because these countries are particularly involved in CMV management. We agree that our review could not provide any conclusive new information because the data are variable and scattered. However, we believe that their practices may be representative and followed in countries with similar epidemiology and health care resources.

We completed our discussion considering his remarks (**lines 379-382**).
